# The Role of Pharmacists in General Practice in Asthma Management: A Pilot Study

**DOI:** 10.3390/pharmacy6040114

**Published:** 2018-10-15

**Authors:** Louise S. Deeks, Sam Kosari, Katja Boom, Gregory M. Peterson, Aaron Maina, Ravi Sharma, Mark Naunton

**Affiliations:** 1 Discipline of Pharmacy, Faculty of Health, University of Canberra, Bruce, Canberra, ACT 2601, Australia; louise.deeks@canberra.edu.au (L.S.D.); katjaboom@hotmail.com (K.B.); g.peterson@utas.edu.au (G.M.P.); aaron.maina@gmail.com (A.M.); mark.naunton@canberra.edu.au (M.N.); 2 Faculty of Health, University of Tasmania, Hobart, TAS 7001, Australia; 3 UCL School of Pharmacy, London WC1N 1AX, UK; ravi.sharma4@nhs.net

**Keywords:** primary health care, asthma, general practice, pharmacists, patient education

## Abstract

**Background**: Asthma is principally managed in general practice. Appropriate prescribing and medication use are essential, so general practice pharmacists appear suitable to conduct asthma management consultations. This pilot study aimed to evaluate the asthma management role of a pharmacist in general practice. **Methods**: Analysis of an activity diary and stakeholder interviews were conducted to identify interventions in asthma management; determine whether asthma control changed following pharmacist input; and determine acceptability of asthma management review by a pharmacist in one general practice in Canberra, Australia. **Results**: Over 13 months, the pharmacist saw 136 individual patients. The most common activities were asthma control assessment; recommendations to adjust medication or device; counselling on correct device use; asthma action plan development and trigger avoidance. For patients with multiple consultations, the mean Asthma Control Test score improved from the initial to last visit (14.4 ± 5.2 vs. 19.3 ± 4.7, *n* = 23, *p* < 0.0001). Eight of the 19 (42%) patients moved from having poor to well-controlled asthma. Case studies and qualitative data indicated probable hospital admission avoidance and stakeholder acceptability of asthma management by a practice pharmacist. **Conclusions**: This pilot study demonstrated it is feasible, acceptable and potentially beneficial to have a general practice pharmacist involved in asthma management. Fuller evaluation is warranted.

## 1. Introduction

Asthma is a common chronic condition that is principally managed in general practice. A 2014–2015 survey determined that of the 10.8% of Australians who had asthma, almost two-thirds (60.9%) had consulted a general practitioner (GP) in the previous 12 months for their condition and 6.0% had consulted a specialist [1]. However, one in four of the asthma patients surveyed reported poor symptom control over the previous 12 months [1], so there may be scope for improvement of asthma management in general practice. Reasons identified for possible suboptimal management included variable adherence to prescribing guidelines and poor utilisation of asthma action plans [2]. Improved asthma control is associated with better quality of life, increased productivity and savings to the health system [3].

Incorrect medication selection and use can lead to poor asthma management and adverse outcomes [4,5,6]. Pharmacists have demonstrated that they can improve asthma control through increasing the appropriate use of medication [7]. Given that most of the asthma care is delivered in general practice, pharmacists in this setting may provide an opportunity to work collaboratively with GPs, nurses and other healthcare professionals to improve asthma management. While not as developed as in the United Kingdom, the role of pharmacists in general practice is becoming more established in Australia. This was recognized by the Australian Government, who announced some funding via The Workforce Incentive Program in 2018 [8]. It was identified that one of the practice pharmacists’ roles would be supporting patients with chronic health conditions [8]. This is consistent with other countries such as England [9]. The National Health Service (NHS) in England states that one of the aims of the clinical pharmacist in general practice is to manage patients with long-term conditions [9].

General practices in Australia are encouraged to engage with asthma patients via the Asthma Cycle of Care, a Medicare Benefits Schedule (MBS) claimable activity for GPs. This comprises two asthma-related consultations within a 12-month period for patients diagnosed with moderate to severe asthma. An Asthma Cycle of Care requires a documented diagnosis, assessment of asthma control and severity, a review of the patient’s use and access to asthma medication and devices, development and subsequent review of a written asthma action plan, and asthma self-management education [10]. The severity of asthma at a point in time can be determined by administering the Asthma Control Test (ACT). The ACT is a patient questionnaire about symptoms, medication use, quality of life and perceived control over the previous 4 weeks. Each of the five questions is assigned a minimum score of 1 and a maximum score of 5, providing a total score between 5 and 25. An ACT score of 19 or below indicates that asthma management needs improvement, while 20 or more indicates good control [11,12].

Practice nurses usually conduct the Asthma Cycle of Care activities, but pharmacists also possess the necessary skills. The aim of this pilot study was to describe interventions in asthma management by one general practice pharmacist, subsequent changes in asthma control and the acceptability of this model of care. This study was nested within a trial conducted in the Australian Capital Territory, Australia [13,14], which was funded by the Capital Health Network, the local Primary Health Network.

## 2. Materials and Methods

Five part-time (15.2–16 h per week) non-dispensing pharmacists were employed by three general practices in the Australian Capital Territory from February 2016. The three practices recruited their own pharmacists without any involvement of the research team, and they had not previously had a pharmacist within the practice. The pharmacists were subsequently utilized according to their own individual skillset and local workplace needs, as independently determined by each practice. Each general practice pharmacist maintained a daily activity diary, the analysis of which has been described elsewhere [13]. Stakeholder experiences with the pharmacists in general practice have also been published [14].

A sub-analysis of the activity diary from one of the pharmacists, who had selected asthma management as an area of focus, and the stakeholder perspectives were conducted to identify which interventions were conducted; determine whether asthma control had improved following pharmacist input, by comparing ACT results at the initial consultation with those at following consultations; and to determine the acceptability of a general practice pharmacist in asthma management.

The initial sub-analysis of the activity diary was conducted by one researcher (AM) and checked by two other researchers (LD, SK). Changes in ACT scores (first to last consultation) were compared for each individual using a paired t-test, while the Fisher’s exact test was used to examine changes in the distribution of patients across categories of asthma control.

Feedback about pharmacist-provided asthma care was obtained from the semi-structured interviews that were conducted by one researcher (LD) with patients, GPs and the practice pharmacist. These interviews were audio-recorded and transcribed verbatim by a professional transcribing agency, with LD checking the transcripts for accuracy. The transcripts were imported into NVivo (version 10.2.2, QSR, Melbourne, VIC, Australia). Two researchers (LD, SK) conducted the thematic analysis by reading the transcripts and adding codes to the data to identify emerging themes that described the data. Each researcher worked independently initially to reduce any bias, then agreed on emerging themes and resolved any discrepancies by discussion.

Case studies were identified in consultation with the practice pharmacist. The University of Canberra Human Ethics Research Committee approved the study (Project number 15-235).

## 3. Results

### 3.1. Activity Diary

Activity diary data was collected from 23 May until 25 November, 2016 and from 15 May until 15 December, 2017. Patients with asthma were either identified and invited to attend for review by the practice pharmacist or referred to the practice pharmacist by the GPs or other members of practice staff. GPs, nurses and receptionists suggested that patients consult with the practice pharmacist if they had concerns about asthma control, compliance with inhalation devices, device selection or requirements for training in device use. The patients’ ages ranged from 17 months to 84 years.

#### 3.1.1. Interventions by the Pharmacist

Overall, 87.5% (119/136) patients who consulted with the practice pharmacist had their asthma control recorded on their first visit and, in general, they had relatively poor asthma control (mean ACT score of 18 ± 5.3; Table 1). The pharmacist conducted activities such as issuing asthma action plans, educating patients, recommending to step up/down therapy, reviewing inhaler technique and making other relevant recommendations such as device changes (e.g., dry-powder to metered-dose inhaler). Step up of therapy comprised increasing or starting corticosteroid/long-acting beta agonist combination inhaler; corticosteroid inhaler; short-acting muscarinic antagonist inhaler; oral corticosteroid; oral montelukast; or long-acting muscarinic antagonist inhaler. Step down of therapy comprised reducing or stopping corticosteroid/long-acting beta agonist combination inhaler; corticosteroid inhaler; or long-acting muscarinic antagonist inhaler. Asthma plans were provided and discussed with all patients. The pharmacist made comprehensive entries into the patients’ clinical notes and discussed prescribing recommendations, the ACT results and the asthma plans with each patient’s GP during or as soon as possible after the consultation. Follow up consultations were arranged with the GP, pharmacist or nurse to monitor progress with alterations to management where necessary. Data from the practice pharmacist asthma consultations is shown in Table 1. Illustrative case studies are provided in Appendix A.

For patients below 6 years of age, the pharmacist mainly talked with the accompanying adult, but involved the children as much as possible. The pharmacist tried to make it fun and engaging for the child e.g. showed pictures or used drawings. The pharmacist also gave stickers that could be placed on inhalers if the child answered questions correctly. Educational games/videos/apps were used for different age groups. Teenagers were usually given an emergency spacer (flat paper spacer) for when they were going out.

#### 3.1.2. Changes in ACT Scores Following the Pharmacist’s Interventions

A subset of 26 patients (19.1%) were seen more than once by the pharmacist during the data collection periods. Following the pharmacist’s interventions, ACT scores improved for 19 patients, worsened for three patients, were unchanged for one patient and data was incomplete for three patients. The mean ACT score for this subset of patients was 14.4 (SD = 5.2) at the first visit and 19.3 (SD = 4.7) at the most recent visit (*n* = 23, t = 5.43, *p* < 0.0001). Prior to the pharmacist review, 17% (4/23) patients had good asthma control (ACT > 19), increasing to 52% (12/23) afterwards (Fisher’s exact test *p* = 0.03). In total, 42% (8/19) patients with poor control moved to well-controlled asthma following review by the practice pharmacist.

### 3.2. Perceptions of Asthma Care Provided by Practice Pharmacist

The themes and data demonstrated that asthma care by the practice pharmacist was acceptable to patients, GPs and the practice pharmacist (see Table 2). At least one avoided hospital admission was attributed to the practice pharmacist’s interventions. The GPs also reported that the practice pharmacist had educated them about asthma.

## 4. Discussion

### 4.1. Summary

This pilot study demonstrated that it is feasible, acceptable and beneficial to have a pharmacist in general practice focusing on asthma management. Following practice pharmacist review, 42% (8/19) of patients moved from poor to well-controlled asthma, while case studies and qualitative data indicated probable hospital admission avoidance and acceptability to stakeholders. The GPs had not previously routinely issued asthma action plans or recorded asthma control scores for patients, so this was an added benefit of pharmacist involvement.

### 4.2. Strengths and Limitations

Data from one pharmacist was analyzed, so all patients received a comparable level of care and completion of the ACT was consistent. The practice pharmacist was a generalist and not an asthma specialist; this can be perceived as advantageous, being more representative of likely future care models. On the other hand, the pharmacist had additional medication review qualifications [15] and was experienced.

We were reliant on the pharmacist accurately self-reporting activities. The general practice population was from one city, so may not be representative of the wider Australian population. In some countries, pharmacists are permitted to prescribe medication but this is not the case in Australia, where this study was conducted; findings may have been different had there been pharmacist prescribing. We acknowledge that the improvements in asthma control in our pilot study may have been affected by other factors, such as seasonal variability of asthma triggers or interventions by other health care professionals. The nature of the study meant there was no control group, although previous studies with a control group have demonstrated pharmacist effectiveness in improving asthma symptoms [16,17].

### 4.3. Comparison with Existing Literature

Improvements in asthma clinical markers in this study are consistent with the improved clinical indicators following practice pharmacist intervention in other chronic conditions [18], and with that found in a systematic review of pharmacists’ effect on asthma control in settings not including general practice [7]. An English study demonstrated that a specialist respiratory pharmacist visiting a general practice could reduce asthma exacerbations and associated costs [19]. In addition, Gums et al. reported that a physician–pharmacist collaborative asthma care model in USA primary care medical offices reduced asthma-related emergency department visits and hospitalizations, and improved both asthma control and quality of life [20].

All the patients who saw the pharmacist received asthma action plans, an intervention recommended by the Australian asthma guidelines [21]. The use of written action plans to enable patients to adjust their medication seems to be more effective in improving health outcomes than other self-management strategies [22]; however, quality evidence is lacking [23]. A project conducted in Minnesota urban community clinics resulted in more patients having optimal asthma control (ACT ≥ 20), by increasing completion of asthma action plans and educating patients about using the plan [24]. This contrasts to the findings of a review in 2017, which concluded that asthma action plans have no effect on hospital attendance due to asthma, asthma symptom scores or adverse events [23].

Good healthcare teamwork in non-acute settings has been linked with better patient impact [25]. The practice pharmacist in this pilot study managed asthma within a multidisciplinary team to achieve shared goals. This approach is recommended in the Australian Asthma Handbook, ‘effective asthma management involves the whole primary care team, working with the person and also their family or carer where appropriate’ [21]. Gums et al. determined that pharmacist and doctor collaboration can improve asthma outcomes [20] and this is consistent with our findings.

Lack of adherence to prescribing guidelines is also associated with poor asthma control [2]. The pharmacist in this study made recommendations using prescribing guidelines. Pharmacists have demonstrated that they can enhance adherence to guidelines in other conditions (e.g. type 2 diabetes and heart failure) [26,27], so it is reasonable to suggest that practice pharmacists may improve adherence to asthma prescribing guidelines.

### 4.4. Implications for Research

Future well-designed studies are required to confirm the promising results reported here and extend to outcome measures such as hospital admissions, quality of life and cost-effectiveness.

## Figures and Tables

**Table 1:** Summarised data from the practice pharmacist asthma consultations.

	Consultations	ACT Values (Mean ± SD)
Number of consultations	166	
Number of patients	136	
Age (years): mean ± SD	33 ± 25 *	
Gender of patients	87 females, 47 males *	
Number of consultations with asthma action plan issued or reviewed	144 (86.7%)	
Number of patients with ACT score recorded on first visit	119 (87.5%)	18 ± 5.3
Number of consultations where step down of therapy was recommended	25 (15.1%)	21 ± 4.0
Number of consultations where step up of therapy was recommended	37 (22.3%)	14 ± 5.2
Number of consultations where device change was recommended	22 (13.3%)	
Number of consultations where spacer was added	40 (24.1%)	
Number of consultations where advice about allergy management was given	14 (8.4%)	
Number of consultations where advice about managing adverse drug reactions was given	12 (7.2%)	
Number of consultations where smoking cessation advice was given	7 (4.2%; all the smokers in the cohort)	
Number of consultations where other interventions were made ( *n*=/ < 5 for each)	exercise(5), thunderstorm asthma(3), referral for bone mineral density measurement(2), recommending influenza vaccination(2), influenza vaccine administration(1), peak flow monitoring(1), sleep hygiene advice(1), spirometry testing(1), prioritising treatment due to cost of medications(1)	

* 2 not specified.

**Table 2:** The perceptions of asthma care provided by practice pharmacist.

Themes	Illustrative Quotes
Satisfaction of patients, GPs and pharmacists	*“They actually made some adjustment—well the GP had made some adjustment to my medication and both of them want to review that together with me and see how that actually goes and whether that sort of caused much of a change in the way in which my breathing with my asthma has actually improved a lot”.* (Patient, 2016) *“One of the most satisfying things is also the asthma cycle of care. Seeing them coming back you look at their asthma, you give their education, they’re coming back and they’re saying they’re feeling so much better”.* (Practice Pharmacist, 2016) *“I think some of the patients have really valued that and got better asthma control so that has been really good”*. (GP2, 2018) *“Patients who have poor adherence that I wasn’t aware of and their asthma is so much better now because they’re actually taking their preventers, which is a revelation to them. I think patients have found the interactions with [the pharmacist] very helpful around asthma”.* (GP1, 2016) *“One’s [patients] that have come in for asthma reviews have been very pleased”.* (GP1, 2018)
Improved care and avoided hospital admission	*“There is a one asthma patient that I have had who was really difficult and basically tolerated really poorly controlled asthma. She would only come in when she was very bad. Again, [the pharmacist] got her in and emphasised the preventative part of it, and I think that’s prevented even hospital admissions as well as improved the lady’s quality of life”.* (GP1, 2018)
Pharmacist as an expert in asthma care	*“I think that whole process has really sharpened me up in terms of the kinds of ways that we do provide this asthma cycle of care. She’s providing extra knowledge into that space and I guess it’s helping the patients with their inhaler technique. She’s taught me some things about inhaler techniques that I didn’t know”.* (GP1, 2016) *“If somebody comes in for asthma, everybody, even the reception, think about me”.* (Practice Pharmacist, 2018)

## Appendix A.1. Case Study One

The pharmacist saw a 4-year-old boy and his mother for a GP-initiated asthma review. His medical history included hayfever and eczema. His medication use included: fluticasone 50 mg metered dose inhaler two puffs twice a day via spacer, montelukast 4mg once a day, salbutamol 100 mg metered dose inhaler two puffs when needed for breathlessness via spacer. The patient’s mother reported that her son had an asthma exacerbation the previous week, probably caused by a cold. She administered montelukast 4 mg daily for 3 days and then stopped because her son had become aggressive and had started having tantrums. The pharmacist explained that behavioral changes can be a rare side effect from the montelukast. Following discussion with the GP, another trial of montelukast was recommended when the child’s asthma was well-controlled. Over the course of a week, the boy’s asthma improved (ACT 11/25 to 19/25). The pharmacist counselled the patient and his mother about asthma medication, triggers and symptoms. One of the identified triggers was uncontrolled hayfever with nasal congestion being his main symptom. The pharmacist suggested a trial of a beclomethasone nasal spray and advised the boy and his mother about limiting time outdoors when the pollen count was high. Asthma inhaler technique was optimised, and written information about asthma and inhaler technique was provided. The mother was unaware of the need to shake the inhaler prior to use, this was reinforced by the pharmacist. The patient and his mother were counselled about the asthma action plan written by the pharmacist, including that if asthma worsens then to start fluticasone.

## Appendix A.2. Case Study Two

A 46-year-old female had attended the hospital emergency department (ED) with an asthma exacerbation one week before her unscheduled initial pharmacist consultation. Her medications were salbutamol CFC-Free 100 mg/dose inhaler 2–6 puffs four times a day and when needed, ipratropium 21 mg/dose inhaler 4 puffs up to four times a day, fluticasone/eformoterol 250/10 inhaler 2 puffs twice a day. Her ACT score was 6/25 at review. The pharmacist provided education about asthma symptoms, discussed possible triggers and provided medication counselling using the lay terms ‘reliever’ and ‘preventer’. The pharmacist optimised inhaler technique and provided a GP-approved asthma action plan. The patient was reviewed by the pharmacist one week later and her ACT score had improved by 7 points (13/25). The pharmacist recommended that salbutamol (100 microgram/dose) be reduced from 6 to 4 puffs four times a day and when needed, with the plan to reduce further as asthma control improved. The pharmacist reinforced the importance of continuing with twice daily use of the steroid inhaler. The asthma action plan was adjusted accordingly.

Three weeks later the patient presented to the pharmacist with worsening of asthma (ACT 7/25). Following refusal to attend ED, the pharmacist collaborated with the GP to initiate oral prednisolone.

Since the pharmacist involvement in her asthma care there have been no hospital attendances; whereas in the same timeframe during the previous year there were two asthma-related ED attendances.

## Appendix A.3. Case Study Three

A 62-year-old male had a practice pharmacist appointment for a chronic obstructive pulmonary disease (COPD) review. He was an ex-smoker with no other chronic conditions. His current medications were tiotropium dry powder inhaler 18 mg daily, indacterol dry powder inhaler 150 micrograms daily, and salbutamol 100 mg 2 puffs when needed for breathlessness. The pharmacist suspected that the patient may have undiagnosed asthma. The pharmacist administered the COPD assessment test (CAT) [28] with the patient. The COPD CAT assesses current health status relating to COPD and impact on quality of life. It is eight questions, each with scores of 0–5, and the maximum total score of 40 [29]. The COPD CAT score relates to impact of COPD and are considered as very high (> 30), high (> 20), medium (10–20) and low (< 10) [29]. The COPD CAT score for the patient was 21. He was experiencing shortness of breath (SOB) and had disrupted sleep. His triggers were listed as cold and flu but the SOB also worsened when he was exposed to dust at work. In consultation with the GP, the practice pharmacist advised the patient to trial an inhaled corticosteroid, ciclesonide 80 mg metered dose inhaler, 2 puffs daily using a spacer. The practice pharmacist counselled the patient on his new medication and inhaler technique. The pharmacist wrote an asthma action plan and explained to the patient how to use the plan. Using the nSpire™ PiKo-6 electronic FEV1/FEV6 meter, the pharmacist assessed respiratory function prior to commencement of the inhaled corticosteroid using the patient’s height (170 cm) to determine predicted values (see Table A1).

Two weeks later, the pharmacist reviewed the patient’s response to the inhaled corticosteroid trial (COPD CAT score = 0/40 and asthma ACT = 20/25). His respiratory function tests were repeated (see Table 1). The diagnosis was changed from COPD to asthma as a result of the pharmacist review. In consultation with the GP, the practice pharmacist advised the patient to stop tiotropium and later indacterol. The asthma action plan was adjusted by the pharmacist accordingly. The pharmacist and patient discussed asthma symptoms, triggers and inhalers.

**Table A1:** Respiratory function test results.

Test	Predicted	Pre Corticosteroid (% Predicted)	Post Corticosteroid (% Predicted)
FEV1	3.01	2.22 (73%)	3.29 (109%)
FEV6	3.75	3.05 (81%)	4.21 (112%)
FEV1/FEV6		0.73	0.78
PEFR		471	469

FEV1 is forced expiratory volume in 1 s; FEV6 is forced expiratory volume in 6 s (used as an alternative to forced vital capacity); PEFR is peak expiratory flow rate.
